# Substance P Induces Rapid and Transient Membrane Blebbing in U373MG Cells in a p21-Activated Kinase-Dependent Manner

**DOI:** 10.1371/journal.pone.0025332

**Published:** 2011-09-23

**Authors:** John Meshki, Steven D. Douglas, Mingyue Hu, Susan E. Leeman, Florin Tuluc

**Affiliations:** 1 Division of Allergy and Immunology, The Children's Hospital of Philadelphia Research Institute, Philadelphia, Pennsylvania, United States of America; 2 Department of Pediatrics, University of Pennsylvania Medical School, Philadelphia, Pennsylvania, United States of America; 3 Flow Cytometry Core Laboratory, The Children's Hospital of Philadelphia Research Institute, Philadelphia, Pennsylvania, United States of America; 4 Department of Pharmacology and Experimental Therapeutics, Boston University School of Medicine, Boston, Massachusetts, United States of America; University of Chicago, United States of America

## Abstract

U373MG astrocytoma cells endogenously express the full-length neurokinin 1 receptor (NK1R). Substance P (SP), the natural ligand for NK1R, triggers rapid and transient membrane blebbing and we report that these morphological changes have different dynamics and intracellular signaling as compared to the changes that we have previously described in HEK293-NK1R cells. In both cell lines, the SP-induced morphological changes are Gq-independent, and they require the Rho, Rho-associated coiled-coil kinase (ROCK) signaling pathway. Using confocal microscopy we have demonstrated that tubulin is phosphorylated subsequent to cell stimulation with SP and that tubulin accumulates inside the blebs. Colchicine, a tubulin polymerization inhibitor, blocked SP-induced blebbing in U373MG but not in HEK293-NK1R cells. Although p21-activated kinase (PAK) is expressed in both cell lines, SP induced rapid phosphorylation of PAK in U373MG, but failed to phosphorylate PAK in HEK293-NK1R cells. The cell-permeable Rho inhibitor C3 transferase inhibited SP-induced PAK phosphorylation, but the ROCK inhibitor Y27632 had no effect on PAK phosphorylation, suggesting that Rho activates PAK in a ROCK-independent manner. Our study demonstrates that SP triggers rapid changes in cell morphology mediated by distinct intracellular signaling mechanisms in U373MG *versus* HEK293-NK1R cells.

## Introduction

Substance P (SP) is a member of the tachykinin family of neuropeptides and it is best known as a neurotransmitter in the central and peripheral nervous systems. SP mediates various CNS functions, including emesis, depression, pain, anxiety and stress [1], [2], [3], [4], [5]. SP has important roles in cancer biology [3], [6], wound healing [7], exocrine gland secretion [8], [9], [10], as well as in neuroendocrine and immune regulation [11], [12], [13], [14], [15].

The effects of SP are mediated by the NK1R, a G-protein coupled receptor which is expressed in many tissues, including the nervous system, the gut, salivary glands, and cells of the immune system [5], [14]. The classical NK1R has a primary structure that includes 407 amino acid residues and is coupled to proteins in the Gq family, leading to phospholipase C activation, intracellular calcium increase and PKC activation [15]. Activated NK1R interacts with β-arrestins, leading to internalization, followed by degradation of the neuropeptide by endothelin-converting enzyme 1, and finally recycling of the receptor [16]. A truncated splice variant of NK1R that lacks 96 amino acid residues at the C-terminus has been described [17], [18], [19], [20], with roles in modulation of responses triggered by cytokines, chemotaxis of macrophages and regulation of HIV replication [15].

We have recently shown that SP induces persistent membrane blebbing in HEK293 cells transfected with full-length, but not with the truncated NK1R [21]. Membrane blebs are dynamic cellular protrusions that form as a result of intense contraction of the actomyosin cell cortex, which consists of a layer of actin, myosin and associated proteins located underneath the cell membrane. The contraction of the actomyosin cortex causes a rapid increase in intracellular hydrostatic pressure that leads to detachment of the lipid bilayer from the cell cortex. In the case of blebbing, membrane expansion is rapid and outpaces typical velocities encountered in protrusions dependent on actin polymerization, such as lamellipodia, and filopodia. Once the bleb is formed, the membrane cortex is built inside the bleb and the actomyosin contraction of the newly formed cortex results in bleb retraction and restoration of the normal cell shape [22].

The implications of blebbing in cell biology are not completely understood, but there is increasing evidence that bleb formation is involved in essential physiological processes. Blebbing occurs during the initial phase of cell spreading on solid substrates and it precedes lamellipodia formation [22]. Tumor cells that migrate through extracellular matrix gels or through connective tissue can use blebbing as an alternative to lamellipodial migration [22]. Embryonic cells in amphibian, fish and *Drosophila melanogaster* can also use bleb-based migration during development [22]. Blebs may also play a key role in polarizing key cellular components that are essential in cell migration [23].

Rho, Rho-associated coiled-coil kinase (ROCK) and myosin light chain kinase (MLCK) are essential in SP-induced cell contraction and formation of membrane blebs in HEK293 cells stably expressing the full-length NK1R [21]. This cellular response triggered by NK1R activation is independent of phospholipase C (PLC), intracellular calcium and protein kinase C – the classical signaling pathways downstream of Gq-coupled receptors.

It has been shown that Rho GTPases are regulated by microtubule dynamics. Nocodazole, a microtubule depolymerizing reagent, induces RhoA activation, in part due to release of GEF-H1, a microtubule-associated RhoGEF [24], [25]. Nocodazole also converts T cells from the classical lamellipodial/uropod migratory phenotype to a blebbing migratory phenotype, which is correlated with increased Rho/ROCK activity [26]. One of the major intracellular signaling molecules involved in the regulation of tubulin function is p21- activated kinase 1 (PAK), which interacts with a variety of other proteins with signaling roles, such as p41Arc, LIMK, merlin, stathmin and MLCK. These interactions generally lead to increased actin and tubulin polymerization [27]. Furthermore, PAK facilitates microtubule formation by phosphorylating tubulin cofactor B [28].

In the present study we show that U373MG astrocytoma cells that endogenously express the full-length NK1R [29] also respond with membrane blebbing when SP is added to medium, but the dynamics of cell morphology changes are distinct from morphological changes we have previously described in HEK293 cells expressing recombinant NK1R.

The aim of this study was to investigate the intracellular signaling mechanisms involved in membrane blebbing in U373MG cells, and to determine the role of tubulin and PAK as key intracellular signaling molecules in cytoskeleton rearrangement mediated by full-length NK1R activation. We have also examined the differences with respect to cell morphology, signaling pathways and potential functional implications between morphological changes triggered by SP in U373MG cells *versus* HEK293 cells.

## Results

### SP triggers morphological changes, intracellular calcium increase and cell impedance changes in U373MG cells

**Figure 1:**
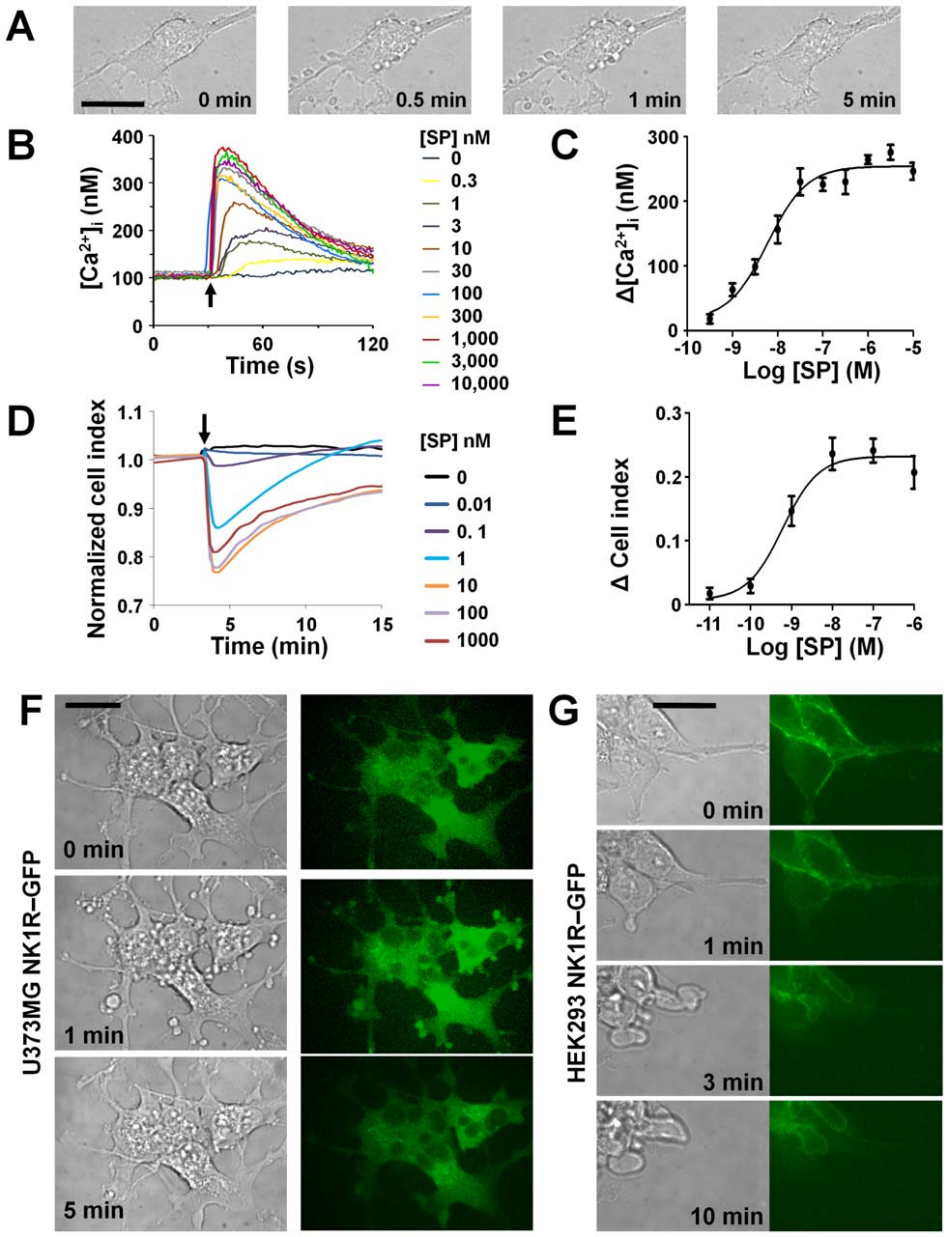
SP causes morphological changes, intracellular calcium increases and impedance changes in U373MG cells. (**A**) Phase-contrast images of U373MG cells treated with SP (100 nM). (**B**) Representative tracings of intracellular calcium in U373MG cells treated with varying concentrations of SP. Addition of SP to medium is indicated by an *arrow*. (**C**) Dose-response curve for the peak intracellular calcium increase induced by SP in U373MG cells. Data were obtained in three independent experiments. (**D**) Representative real time cell electronic sensing (RT-CES) recordings in U373MG cells treated with varying concentrations of SP. Addition of SP to medium is indicated by an *arrow*. (**E**) Dose-response curve for the maximum decrease of impedance signal induced by SP in U373MG cells. Data were obtained in three independent experiments. (**F, G**) DIC and confocal images of U373 NK1R-GFP and HEK293 NK1R-GFP cells stimulated with SP (100 nM). Images were taken at the indicated times. Bars = 20 µm.

Treatment with the endogenous NK1R agonist, SP, caused rapid and transient membrane blebbing in U373MG glioblastoma cells (Fig. 1*A*). Previously we had reported dramatic morphological changes induced by SP in HEK293 transfected with the NK1R receptor. U373MG cells endogenously express this receptor. Although both cell types undergo morphological changes, there are differences between the changes induced by SP in U373MG and those induced in HEK293-NK1R cells. The blebs in U373MG cells started to form in less than 30 seconds, the greatest number of blebs appeared at approximately 1 minute and completely disappeared within several minutes from the addition of SP to the medium. The blebs in the HEK293-NK1R cells lasted for several hours and were much larger than those observed in U373MG cells (Table 1). Binding of SP to full-length NK1R triggers intracellular signaling via the heterotrimeric Gq protein, resulting in transient intracellular calcium increase. SP treatment of U373MG cells results in intracellular calcium increase in a dose-dependent manner with an EC_50_ of 6.0 nM (Fig. 1*B&C*). SP doses of 0.3 nM were sufficient to elicit a measurable increase of intracellular calcium concentrations.

**Table 1:** Characteristics and intracellular signaling requirements for membrane blebbing in U373MG cells *vs.* HEK293 cells.

	HEK293-NK1R	U373MG
Initiation of blebbing	2 min	30 sec
Size of blebs	up to 25 µm	<5 µm
Duration of blebbing	>10 min.	<3 min.
Blebs lead to formation of new adhesion points	Yes	No
Mechanism Gq-dependent	No	No
Mechanism Rho/ROCK-dependent	Yes	Yes
Mechanism tubulin-dependent	No	Yes
Mechanism PAK 1-dependent	No	Yes

We have used the technology developed by Acea Biosciences Inc. (San Diego, CA), to measure the electrical impedance of adherent cells. When the cell number is maintained constant, the impedance change depends mainly on cell interaction with the electrodes. This principle has been successfully used in the past for monitoring the activation of G protein-coupled receptors [30], as well as by our lab to monitor cell morphology changes [21]. SP caused changes in the electric impedance of U373MG cells measured in microtiter plates with built-in electrodes using the RT-CES device and software from Acea Biosciences Inc. (San Diego, CA). Treatment of U373MG cells with varying concentrations of SP caused a rapid and transient dose-dependent decrease in impedance with an EC_50_ of 0.61 nM (Fig. 1*D&E*). The changes in impedance were transient and the maximal decrease occurred at 1 minute after the addition of SP, simultaneously with the maximum bleb formation.

In order to determine whether the differences in bleb formation between U373MG and HEK293 NK1R cells were due to the transfected receptor being expressed at higher levels than the endogenous receptor, we transfected a plasmid encoding the NK1R-GFP fusion protein (Origene, Rockville, MD) into U373MG cells and HEK293 cells. Both U373MG (Fig. 1*F*) and HEK293 cells (Fig. 1*G*) express high levels of NK1R-GFP. Subsequent to cell stimulation with SP (100 nM), membrane blebs that formed in U373MG cells expressing the tagged NK1R displayed a morphology similar to the blebs observed in nontransfected U373MG cells. In HEK293 cells overexpressing the GFP-tagged NK1R, SP induced formation of blebs (Fig. 1*G*) with morphology similar to those that we previously reported in HEK293 overexpressing non-tagged NK1R [21].

### Aprepitant blocks SP-induced blebbing, intracellular calcium increase and impedance changes

**Figure 2:**
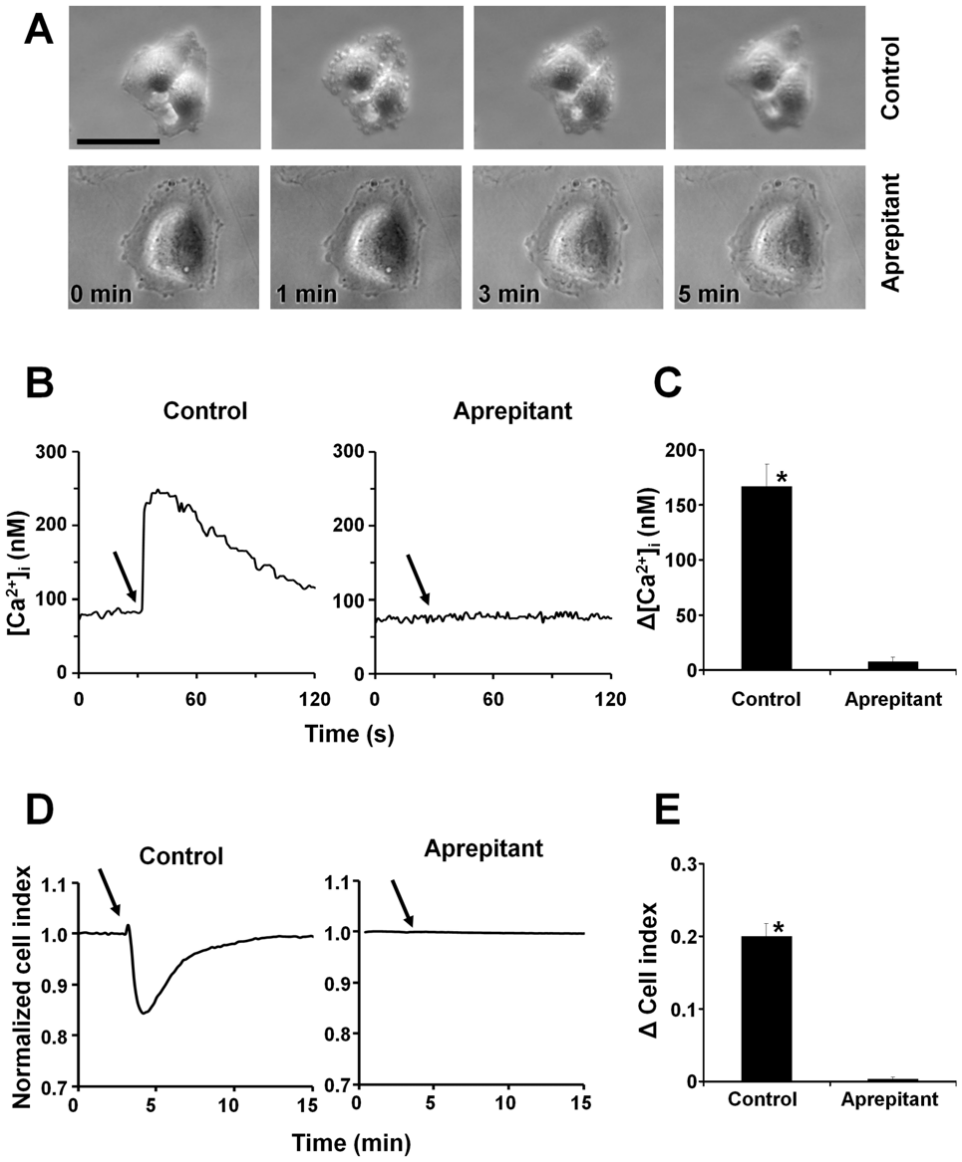
NK1R antagonist, aprepitant blocks SP-induced blebs, intracellular calcium increase and impedance changes. (**A**) Phase-contrast images of live U373MG cells preincubated with control or aprepitant (1 µM) for 10 min and then stimulated with SP (100 nM). (**B**) Representative intracellular calcium recordings of U373MG cells preincubated with *(left)* control or *(right)* aprepitant (1 µM) for 10 min and then stimulated with SP (100 nM) where indicated by *solid arrow*. (**C**) Change in intracellular calcium induced by SP in U373MG cells preincubated with either control or aprepitant (1 µM) for 10 min and then stimulated with SP (100 nM). Data are expressed as mean ± S.E. (error bars) of three independent experiments. (**D**) Representative cell impedance recordings of U373MG cells preincubated with *(left)* control or *(right)* aprepitant (1 µM) then stimulated with SP (100 nM) where indicated by *solid arrow*. Bar = 20 µm. (**E**) Decrease in cell impedance induced by SP in U373MG cells preincubated with either control or aprepitant (1 µM) for 10 min and then stimulated with SP (100 nM). Data are expressed as mean ± SEM of three independent experiments. (* p<0.05).

In order to test whether the effect of SP was mediated by the NK1R receptor rather than *via* a non-specific mechanism, we examined the effect of the selective NK1R antagonist, aprepitant, on SP-induced blebbing, intracellular calcium increase and cell impedance decrease. Phase-contrast microscopy revealed that pretreatment of U373MG cells with aprepitant blocked SP-induced bleb formation (Fig. 2*A* *,* video S1). In accordance with the morphological data, examination of the SP-induced intracellular calcium increase, an early event in the G protein coupled receptor signaling, revealed that aprepitant completely blocked SP-induced intracellular calcium changes (Fig. 2*B&C*). Aprepitant completely blocked the changes in SP-induced cell impedance (Fig. 2*D&E*). These data indicate that the SP-induced effects on U373MG cells are a result of activation of the full-length NK1R.

### Shape Change in U373MG cells is not due to intracellular calcium increase

**Figure 3:**
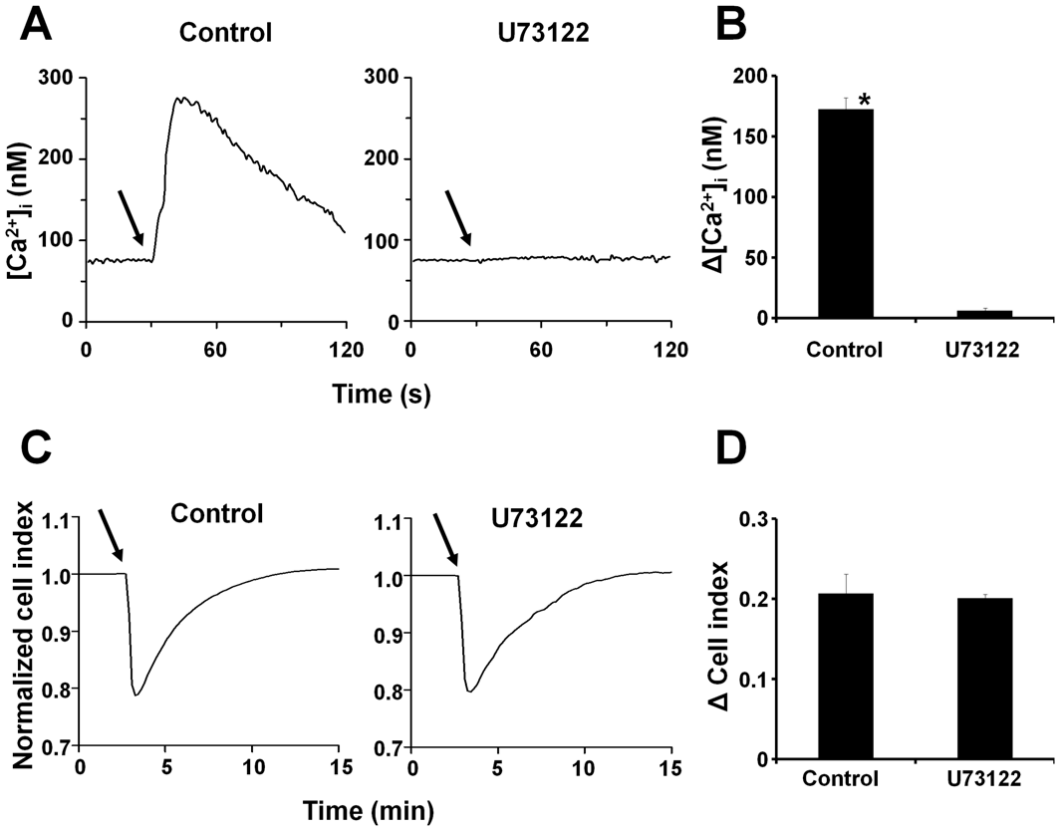
Cellular blebbing induced by NK1R activation is not blocked by the PLC inhibitor U73122. (**A**) Representative intracellular calcium recordings of U373MG cells preincubated with *(left)* control or *(right)* U73122 (10 µM) for 60 min and then stimulated with SP (100 nM) where indicated by *solid arrow*. (**B**) Change in intracellular calcium induced by SP in U373MG cells preincubated with either control or U73122 (10 µM) for 60 min and then stimulated with SP (100 nM). Data are expressed as mean ± S.E. (error bars) of three independent experiments. (**C**) Representative cell impedance recordings of U373MG cells preincubated with *(left)* control or *(right)* U73122 (10 µM) for 60 min and then stimulated with SP (100 nM) where indicated by *solid arrow*. (**D**) Decrease in cell impedance induced by SP in U373MG cells preincubated with either control or U73122 (10 µM) for 60 min and then stimulated with SP (100 nM). Data are expressed as mean ± SEM of three independent experiments. (* p<0.05).

In order to determine whether activation of phospholipase C plays a role in the shape change induced by SP, we treated U373MG cells with the phospholipase C (PLC) inhibitor U73122. Fig. 3*A&B* shows that the U73122 treatment was sufficient to abolish changes in intracellular calcium. In contrast treatment with U73122 did not block SP-induced bleb formation as visualized by phase-contrast microscopy (video S1). Also there was no effect on the changes in impedance caused by SP in U73122-treated U373MG cells, Fig. 3*C&D*.

### Cellular blebbing in U373MG cells induced by NK1R activation is mediated by the Rho/ROCK/MLCK pathway

**Figure 4:**
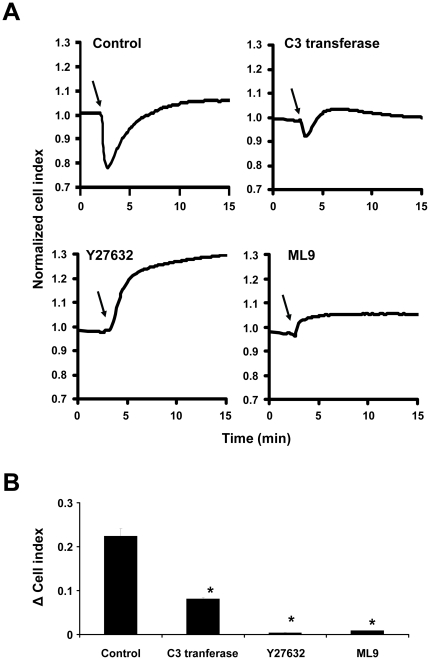
Cellular blebbing in U373MG cells induced by NK1R activation is mediated by the Rho/ROCK/MLCK pathway. (**A**) Representative cell impedance recordings of U373MG cells preincubated with control, 2.0 µg/ml C3 transferase, 10 µM Y27632 or 50 µM ML9 for 60 min and then stimulated with SP (100 nM) where indicated by *solid arrow*. Data are representative of three independent experiments. (**B**) Decrease in cell impedance induced by SP in U373MG cells preincubated with either control, 2.0 µg/ml C3 transferase, 10 µM Y27632 or 50 µM ML9 for 60 min and then stimulated with SP (100 nM). Data are expressed as mean ± SEM of three independent experiments. (* p<0.05).

SP-induced bleb formation in HEK293 NK1R is mediated though activation of the Rho/ROCK/MLCK pathway and is dependent on phosphorylation of MLC [21]. Therefore, we tested the effect of blocking each of these steps on both SP-induced bleb formation and changes in impedance. We used a cell-penetrating form of the *Clostridium botulinum* exoenzyme C3 transferase (Cytoskeleton Inc., Denver, CO) to block the Rho GTPases. Pretreatment of U373MG cells with C3 transferase for 4 h greatly inhibited the SP-induced decrease of impedance values (Fig. 4), and it completely blocked membrane blebbing (video S1). Y27632, a highly specific cell-permeable inhibitor of ROCK, prevented SP-induced blebbing (video S1). The typical SP-induced decrease in cell impedance was also abolished (Fig. 4). Furthermore, cell impedance increased after treatment with SP, which is consistent with our previous findings in HEK293-NK1R cells. This is most likely due to cells spreading on the substrate. U373MG cell treatment with ML-9, an inhibitor of MLCK, completely blocked bleb formation (video S1), as well as SP-induced decrease in impedance (Fig. 4). These data indicate that Rho, ROCK and MLCK are key signaling mediators of SP-induced blebbing in U373MG cells.

### Tubulin is present in the blebs of U373MG and HEK293-NK1R cells

**Figure 5:**
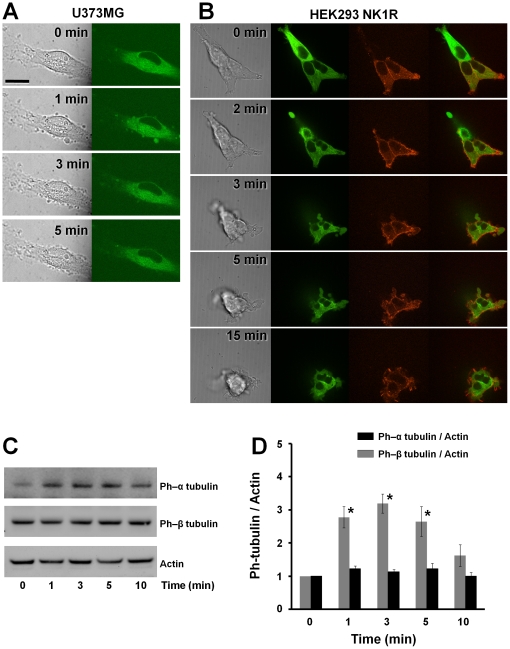
Tubulin is present in the blebs formed in U373MG and HEK293-NK1R cells. DIC and confocal images of (**A**) U373MG cells and (**B**) HEK293-NK1R cells. A plasmid encoding a chimeric protein containing GFP fused to the N terminus of β-tubulin *(green)* was transfected in both cell lines. RFP fused to the N terminus β-actin *(red)* was also expressed in HEK293-NK1R cells. Cells were stimulated with 100 nM of SP and images were taken at the indicated time points. Bar = 20 µm. (**C**) U373MG cells were stimulated with SP (100 nM) and lysed at the indicated times. Cell lysates were analyzed by Western blotting using primary antibodies directed at phospho-α tubulin, phospho-β tubulin and actin. A representative experiment is shown. (**D**) Densitometric analysis of α tubulin and β tubulin phosphorylation in response to SP (100 nM). Data are expressed as mean ± SEM of three independent experiments. (* p<0.05).

In order to investigate whether microtubules are present within and thus influence formation of SP-induced blebs, U373MG and HEK293-NK1R cells were infected with baculovirus encoding human tubulin-GFP and actin-RFP (Invitrogen, Carlsbad, CA). Cells were placed in 96 well plates and SP (100 nM) was added to each well while time-lapse imaging was performed on a spinning disk confocal microscope, as described under Materials and Methods. Both cell types responded with bleb formation following stimulation with SP (Fig. 5). U373MG cells underwent transient blebbing, that was obviously apparent at 1 minute and the blebs disappeared at 3 minutes after the addition of SP. However, the cell membrane had irregular aspect under DIC microscopy (Fig. 5*A*, *left panel* *s,* video S2). HEK293-NK1R cells (Fig. 5*B*) responded with rapid cell body contraction, and some of the cells detached from the substrate but maintained contact with neighboring cells. Membrane blebbing started later and lasted longer than in U373MG cells (Table 1). Furthermore, some blebs established new cell adhesion points to the bottom of the well (video S3). The distribution of the tubulin-GFP inside the blebs was visible in both cell lines. Actin-RFP expression in the U373MG cells was very low (data not shown) and we were not able to take images clearly showing actin distribution within the blebs. In the HEK293-NK1R cells, actin accumulated near the plasma membrane.

Because tubulin is present in the blebs, we examined SP-induced tubulin activation. Phosphorylation of tubulin was examined using a phosphospecific antibody that recognizes tyrosine 272 on α-tubulin and serine 172 on β-tubulin. Treatment with 100 nM of SP induced a significant increase in α-tubulin but not β-tubulin (Fig. 5C&D) in U373MG cells. The phosphorylation was high between 1-3 minutes and began fading after that. This data indicates that SP treatment of U373MG cells resulted in tubulin phosphorylation.

### Tubulin has a key role in SP-induced membrane blebbing in U373MG but not HEK293 NK1R cells

Accumulation of tubulin in SP-induced blebs suggested that tubulin may be an important requirement in cytoskeleton rearrangement triggered by NK1R activation. To test this possibility, we evaluated the effect of colchicine, which prevents assembly of microtubules by unfolding a region of the carboxyl-terminal of β-tubulin, on SP-induced bleb formation in both U373MG and HEK293-NK1R cells. After pretreatment with 2 µM colchicine, U373MG cells had a flat morphology (video S4) and SP treatment failed to cause the formation of membrane blebs. In contrast to U373MG cells, colchicine treatment affected the morphology of the HEK293-NK1R cells in a different manner, causing the cells to appear less adherent and to round up (video S4). Furthermore, colchicine was not able to block the SP-induced blebbing in HEK293-NK1R.

**Figure 6:**
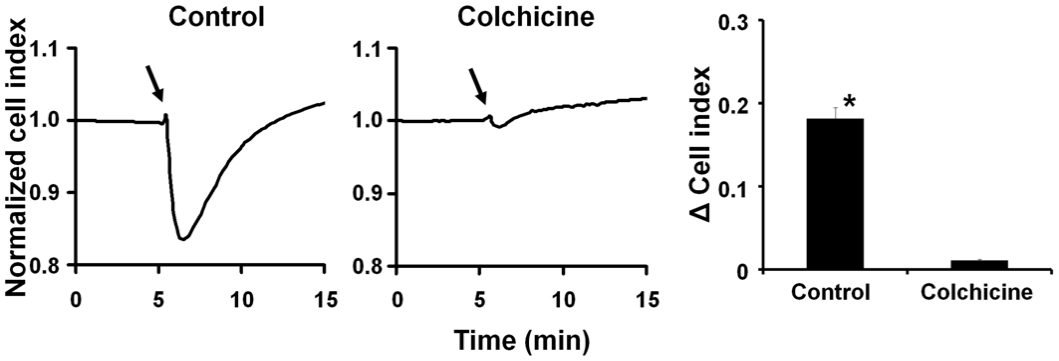
Tubulin has a key role in SP-induced morphological changes in U373MG but not HEK293-NK1R cells. (**A**) Representative cell impedance recordings of U373MG and preincubated with *(left)* control or *(right)* colchicine (10 µM) for 1 hour and then stimulated with SP (100 nM) where indicated by *solid arrow*. (**B**) Decrease in cell impedance induced by SP in U373MG cells preincubated with either control or colchicine (10 µM) for 1 hour and then stimulated with SP (100 nM). Data are expressed as mean ± SEM of three independent experiments. (* p<0.05).

Additionally, colchicine treatment blocked the changes in impedance caused by SP in U373MG (Fig. 6). Because colchicine treatment caused the HEK293-NK1R cells to round up we were not able to measure cell impedance changes for this cell line. These data indicate that normal tubulin polymerization was essential only for bleb formation in U373MG cells, but not in HEK293 cells.

### PAK mediates NK1R induced cellular blebbing in U373MG cells

**Figure 7:**
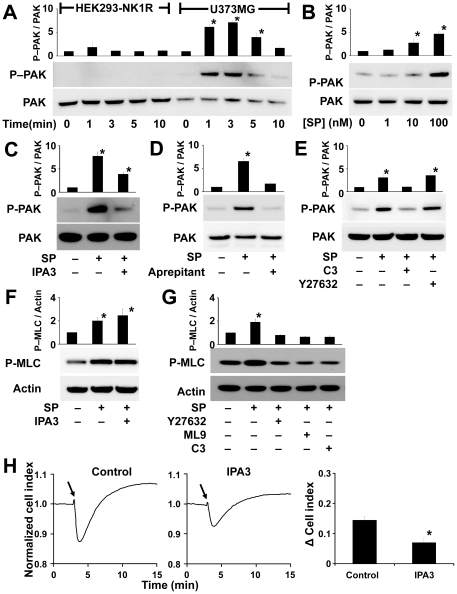
PAK mediates NK1R induced cellular blebbing in U373MG cells. Cell lysates were analyzed by Western blotting using primary antibodies directed at phospho–PAK, PAK, phospho-MLC or actin. Densitometric analysis of (**A**) PAK phosphorylation in U373MG and HEK–NK1R cells stimulated with SP (100 nM) and lysed at the indicated times. (**B**) PAK phosphorylation in U373MG cells stimulated with SP for 1 min at the indicated concentrations. (**C-E**) PAK phosphorylation in U373MG cells preincubated with control, IPA3 (20 µM), aprepitant (1 µM), C3 transferase (2.0 µg/ml), or Y27632 (10 µM) for 1 hour, and then stimulated with SP (100 nM) for 1 min. (**F,G**) MLC phosphorylation in U373MG cells preincubated with control, IPA3 (20 µM), Y27632 (10 µM), ML9 (10 µM), or C3 transferase (2.0 µg/ml), for 1 hour, and then stimulated with SP (100 nM) for 1 min. Data are expressed as mean ± SEM of three independent experiments. A representative experiment is shown below each data set. (**H**) Cell impedance recordings of U373MG cells preincubated with *(left)* control or *(right)* IPA3 (20 µM) for 60 min and then stimulated with SP (100 nM) where indicated by *solid arrow*. Bar = 20 µm. Data are expressed as mean ± SEM of three independent experiments. (* p<0.05).

Because PAK is involved in cytoskeleton rearrangement and microtubule dynamics, we examined SP-induced PAK activation. PAK1 (65 kDa) and PAK2 (62 kDa) are highly homologous PAK family members with autophosphorylation sites on serine residues in the N-terminal regulatory region [31]. Phosphorylation of PAK was examined using a phosphospecific antibody that recognizes serine 199 and 204 on PAK1 and serine 192 and 197 on PAK2 (Fig. 7). Treatment with 100 nM of SP induced a significant increase in PAK phosphorylation in U373MG cells but not HEK293 NK1R cells (Fig. 7*A*). The phosphorylation was intense at 1 min and almost completely disappeared by 10 min. Treatment with 1, 10 and 100 nM SP showed a dose-dependent increase in PAK phosphorylation (Fig. 7*B*). Treatment with 20 µM IPA3 resulted in inhibition of SP-induced PAK phosphorylation (Fig. 7*C*). Next, we pretreated with 1 µM aprepitant which resulted in a complete block of SP-induced PAK phosphorylation (Fig. 7*D*). We tested the effect of blocking the Rho/ROCK pathway on PAK phosphorylation. Treatment with the Rho inhibitor C3 transferase blocked the phosphorylation of PAK (Fig. 7*E*), indicating that Rho may mediate SP-induced PAK phosphorylation. Treatment with the ROCK inhibitor Y27632 had no apparent effect on PAK phosphorylation (Fig. 7*E*), which indicates that Rho was not acting through ROCK to mediate PAK phosphorylation. Because inhibiting Rho had an effect on PAK phosphorylation, we tested whether PAK plays a role on MLC phosphorylation. We used the PAK inhibitor IPA3 in U373MG cells treated with SP and looked at phosphorylation of MLC. Inhibition of PAK did not affect MLC phosphorylation (Fig. 7*F*), indicating that Rho was not acting though PAK to cause MLC phosphorylation. Treatment with Y27632, ML9 and C3 transferase, all resulted in decrease in MLC phosphorylation (Fig. 7*G*). Additionally, we tested the effect of IPA3 (20 µM) on SP-induced impedance changes. Fig. 7*H* shows a significant decrease in the impedance change from 0.14±0.02 to 0.07±0.03. Phase-contrast microscopy imaging of U373MG and HEK293 cells pretreated with IPA3 showed that SP-induced blebbing was greatly inhibited in the U373MG but not in HEK293 cells (video S4). These data indicate that SP treatment of U373MG cells resulted in PAK phosphorylation, and inhibiting PAK caused an obvious inhibition of SP-induced bleb formation.

## Discussion

In the present study we have shown that SP induces rapid formation of small and transient membrane blebs in U373MG cells (Fig. 1). The morphology changes induced by SP in U373MG cells have different aspect and dynamics as compared to those that we have previously described in HEK293 cell [21]. The blebs in U373MG cells are not involved in the formation of membrane ruffles and new adherence areas to solid substrate, as it happens in HEK293-NK1R cells (video S3). Having eliminated the possibility that the differences in NK1R mediated cell shape response in U373MG versus HEK293 cells could not be explained by low expression levels in U373MG, we hypothesized that distinct intracellular signaling mechanisms activated by NK1R in the two cell types are responsible for clearly distinct morphology changes induced by SP in the two cell lines.

The effect of SP on cell impedance in U373MG cells is similar to our previous observations in HEK293-NK1R cells [21]. A major difference between the two cell lines was that the response in U373MG cells was transient, the cells clearly showing a peak decrease in impedance that occurred in about one minute from SP application (Fig. 1*D*). The decrease in impedance value is an indication of cell cortex contraction that results in a smaller area of the microelectrodes being covered by cells. The transient nature of the impedance changes in U373MG cells indicates that the normal adherence to the solid substrates is only minimally affected by persistent stimulation with SP. This is in stark contrast with impedance changes observed in HEK293-NK1R cells, where the persistence of low impedance values indicates that HEK293-NK1R cells undergo more dramatic and persistent morphological changes than U373MG cells.

In order to demonstrate that the effect of SP on U373MG cells is mediated by NK1R we have used aprepitant, a selective NK1R antagonist [32]. We have concluded that the effect of SP is mediated by NK1R, since aprepitant completely blocked SP-induced blebbing, calcium increase and impedance changes (Fig. 2) in U373MG cells.

The PLC inhibitor U73122 blocked intracellular calcium increase caused by SP, proving that PLC was effectively inhibited. However, SP-induced blebbing and cell shape change was not affected, suggesting that these effects are independent the Gq-mediated signaling events which are normally associated with intracellular calcium increase. Furthermore, the EC50 for the impedance response was about 10 times lower than the EC50 for the calcium response, indicating that the morphological changes do not depend on increased intracellular calcium. We have found that SP does not cause significant increase in cAMP levels (data not shown), thus Gs does not mediate the effect of SP. It is likely that the SP-induced effect of cell shape changes involve the participation of other G proteins, such as G12/13, which are known to be involved in cytoskeleton remodeling.

Rho, ROCK and MLCK are key mediators of NK1R induced cellular blebbing in HEK293-NK1R cells [21]. Rho contributes to MLC phosphorylation through the activation of ROCK. ROCK activates MLCK, which phosphorylates MLC and thus activates the actin-myosin machinery necessary for the formation of membrane blebs. The Rho inhibitor C3 transferase, the ROCK inhibitor Y27632, and the MLC kinase inhibitor ML9, all greatly inhibited SP-induced cell impedance decrease in U373MG cells (Fig. 4*A*), and they completely blocked cell blebbing (Fig. 4*B*). These findings are consistent with our previous observations in HEK293-NK1R [21]. Thus, we conclude that Rho/ROCK/MLCK is an essential signaling pathway in membrane blebbing in both U373MG and HEK293 cells.

The role of the Rho/ROCK pathway on microtubules has been demonstrated to be important in the regulation of migratory polarity of T cells [26]. We have demonstrated that GFP-tagged tubulin is present in blebs in both U373MG and HEK293-NK1R cells. Furthermore, we have shown that in U373MG cells SP induces rapid phosphorylation of α-tubulin at the tyrosine-272 residue and this finding also suggests tubulin is involved in the complex intracellular responses mediated by NK1R. We have also shown that actin, which is a major component of cell cortex, concentrates at the periphery of the blebs in HEK293 cells. It has been postulated that actin coats the bleb to create new cell cortex inside the bleb. The newly formed cortex contracts in order to restore the normal shape of the cell [33]. Unlike HEK293 cells which are rather loosely adherent to substrates, the U373MG cells are highly adherent to solid substrates. U373MG cells consistently expressed lower levels of fluorescent proteins than HEK293 cells. This was especially the case of RFP-tagged actin, which U373MG cells did not express at levels sufficient for reliable detection using confocal microscopy.

Colchicine, which prevents curved tubulin of adopting a straight structure required for polymerization [34], has completely different effects on cell blebbing in the two cell lines studied: it blocks SP-induced blebbing in U373MG cells; while it was unable to block bleb formation in HEK293-NK1R cells (video S1). Colchicine inhibited cell impedance changes induced by SP in U373MG cells (Fig. 6). HEK293-NK1R cells showed a tendency to detach, that interfered with impedance measurements.

PAK proteins are a highly conserved group of serine/threonine kinases [35] and they are well characterized effectors of Rac and Cdc42 [35]. PAK has been implicated in proliferation, survival and cancer invasiveness, as well as in the regulation of morphological processes, controlling cell polarity and actin cytoskeleton organization [36], [37], [38]. SP induces phosphorylation of PAK in U373MG but not HEK293 cells. The phosphorylation of PAK occurred simultaneously with membrane blebbing and GFP-tagged tubulin concentration in the blebs. The distinctive morphological changes induced by NK1R activation in U373MG and HEK293 cells may be a consequence of their differential phosphorylation of PAK. PAK activation can occur though many mechanisms: activation by Rho-family GTPases [27], phosphorylation by 3-phosphoinositide-dependent kinase-1 or interaction with lipids [39], activation by G protein-coupled receptor kinase-interacting target 1 (GIT1) independently of the small GTPases [39], [40], Akt [41] and through caspase 3 cleavage [42]. Treatment with the PAK inhibitor, IPA3, had no effect on HEK293- NK1R cell's ability to bleb, which was consistent with the lack of PAK phosphorylation. Treatment with IPA3 in U373MG cells did abolish their ability to bleb. There are numerous reports that link PAK activation to microtubule assembly. Micro-injection of activated PAK1 in Swiss 3T3 cells caused the formation of lamellipodia, filopodia and membrane ruffles [43]. PAK promotes microtubule assembly by phosphorylating and inactivating the MT-associated protein Op18/Stathmin [44], and PAK1 regulates microtubule dynamics by phosphorylating tubulin cofactor B [28].

The Rho inhibitor C3 transferase blocked NK1R-mediated PAK phosphorylation, indicating that Rho is mediating the activation of PAK. The fact that treatment with the ROCK inhibitor Y27632 had no effect on PAK phosphorylation indicates that PAK activation is independent of ROCK. Rho is thus acting on ROCK to mediate changes in actin contractility and on PAK to mediate tubulin polymerization.

We have demonstrated that SP induces distinct morphological changes that are mediated by NK1R and these changes are dependent on the cell host. We have found major differences between membrane blebbing in U373MG cells and HEK293 cells expressing the NK1R receptor. SP-induced blebbing in HEK293 cells is more intense and persistent and cells use the blebs to form new adherence points to the solid substrate. In U373MG cells blebbing lasts only for a few minutes and the blebs disappear without modifying cell shape. In both cell types the activation of Rho/ROCK/MLCK signaling pathway is essential for triggering cell blebbing. However, in U373MG cells the membrane blebbing is associated with dual activation of the Rho/ROCK/MLCK pathway and PAK activation. Furthermore, in U373MG cells blebbing is associated with α-tubulin phosphorylation.

**Figure 8:**
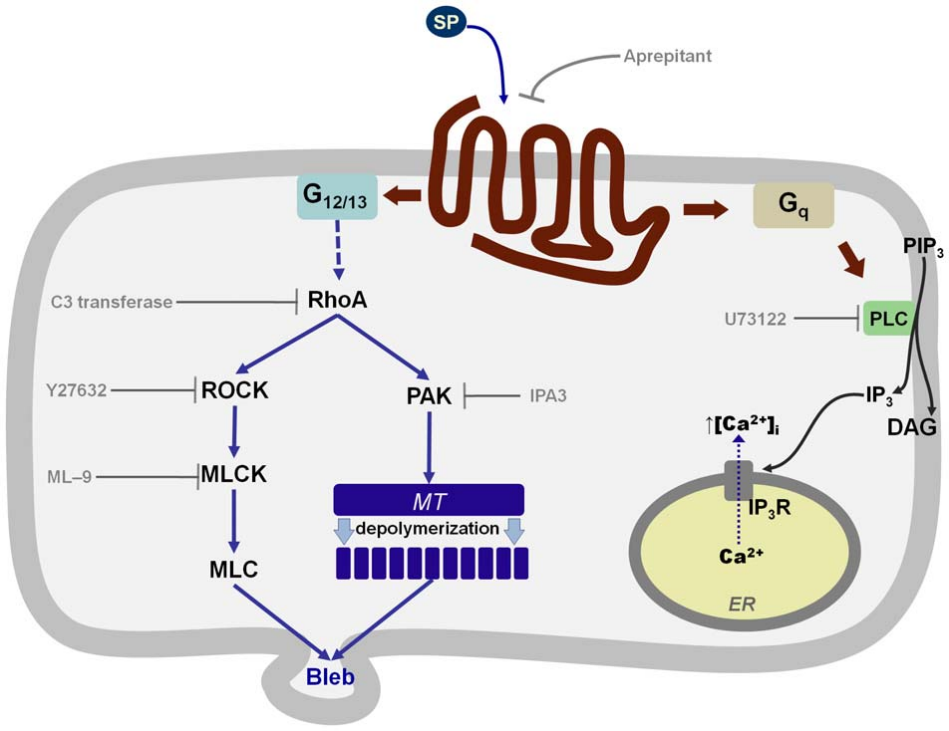
Intracellular signaling mechanisms involved in NK1R-mediated membrane blebbing. Bleb formation mediated by the endogenous full-length NK1R in U373MG cells is dependent of PAK and is associated with tubulin depolymerization. The classic Gq-dependent signaling pathway triggered by NK1R is not involved in morphological changes induced by SP.

A schematic model summarizing the signaling mechanisms implicated in cell morphology changes induced by SP in U373MG cells is depicted in Fig. 8.

Thus, our study demonstrates that SP triggers complex and heterogeneous changes of cell morphology which are mediated by well-defined intracellular signaling mechanisms. The full spectrum of implications of NK1R-mediated cellular shape change in cell physiology and in pathology has yet to be elucidated. The blebs may also serve to aid fusion of HIV particles with the host plasma membrane [45], [46]. Multiple studies have demonstrated that membrane blebbing is an important mechanism of migration of cancer cells [26], [47], [48] and understanding the functional implications and the signaling mechanisms involved in this process may lead to the development of novel drugs that are able to interfere with cancer cell invasion.

## Materials and Methods

### Reagents

Substance P and ML-9 were purchased from Sigma (St. Louis, MO). C3 transferase was from Cytoskeleton Inc. (Denver, CO). Y27632, colchicine, and U73122 were from Biomol (Plymouth Meeting, PA). 1, 1′-Dithiodi-2-naphthtol (IPA3) was from Tocris Bioscience (Ellisville, Missouri). GF109203X was from Calbiochem (Gibbstown, NJ). The NK1R antagonist, aprepitant (Emend®), manufactured by Merck & Co. (Whitehouse Station, NJ), was purchased through the Children's Hospital of Philadelphia Pharmacy and purified by chromatography.

*GFP-NK1R*. The plasmid encoding GFP-tagged ORF clone of Homo sapiens tachykinin receptor 1 (TACR1), (accession number NM_001058.2) was purchased from Origene (Rockville, MD).

### Cell Lines

HEK293 and U373MG astrocytoma cells (American Type Culture Collection, Rockville, MD) were cultured at 37°C and 5% CO_2_ in 100-mm cell-culture dishes (Fisher Scientific, Pittsburgh, PA) in Dulbecco's modified Eagle's medium (DMEM) containing 4.5 g/L glucose, 584 mg/L L-glutamine & 110 mg/L sodium pyruvate (Mediatech, Manassas, VA), supplemented with 100 U/ml penicillin, 100 µg/ml streptomycin (Invitrogen, Carlsbad, CA), 100 nM MEM Non-Essential Amino Acids Solution and 10% fetal bovine serum (Hyclone, Logan, UT).

### SP-induced calcium mobilization in U373MG cells

Intracellular calcium measurements were performed in fura-2 loaded cells using Tsien's ratiometric method [31] as previously described [21]. Briefly, cells were seeded in 96-well plates and allowed to adhere overnight in a standard cell culture incubator, at 37°C in a humidified atmosphere of 5% carbon dioxide and air. Fura-2 loading was performed by incubating cells for 45 minutes at room temperature in medium containing 4 µM fura 2-AM and 0.01% Pluronic F-127 (Molecular Probes, Eugene, OR). Cells were washed in Hank's balanced salt solution (HBSS) containing 1 mM calcium chloride and fluorescence was recorded in individual cells using an imaging system from Photon Technologies Inc. (Lawrenceville, NJ). Recordings were performed using excitation wavelengths of 340 and 380 nm and emitted light was measured at 510 nm. The ratio of emitted fluorescence intensity 340/380 nm was used to assess the relative increase in intracellular calcium concentration.

### Expression of GFP-Tubulin and RFP-actin

Cells were infected with baculovirus encoding human tubulin fused to green fluorescent protein (GFP) or actin fused to red fluorescent protein (RFP) (Cellular Lights^TM^ GFP-Tubulin, Invitrogen) as per the manufacturer's instruction. Briefly, cells were plated at a density of 70,000 cells per well of a 24-well plate. After 24 hours the cells were then incubated with the Cellular Lights^TM^ reagent for 2 hours in the dark with gentle shaking. The cells were then treated with enhancer solution for 2 hours at 37°C. Finally the cells were incubated in the complete DMEM solution listed above.

### Microscopy

Cells were placed in 24- or 96-well plates and allowed to adhere overnight by incubation in complete DMEM at 37°C, 5% CO_2_. Medium was replaced with HBSS containing calcium and magnesium and either 100 µl solvent or SP was added to one well at a time while images were acquired. Where indicated, cells were incubated with antagonists or inhibitors prior to addition of substance P. *Phase contrast and fluorescence microscopy images* were acquired on a system including an Olympus IX51 microscope (Olympus America, Center Valley, PA), a Lambda LS 300W Xenon lamp, an external filter wheel and shutter, Lambda 10B controller (Sutter Instrument, Novato, CA), and Hamamatsu Orca C8484-03G02 digital camera (Hamamatsu Corp., Bridgewater, NJ). Dichroic interference contrast (DIC) and c*onfocal microscopy* images were acquired on a Yokogawa CSU 10 spinning disk confocal microscope. The microscope stand was an Olympus IX 71 (60X lens specifications: Olympus 60X, 1.2 NA UPlanSApo water immersion objective 100X lens specification: Olympus 100X, 1.4 NA UPlanSApo oil immersion objective) (Olympus America, Center Valley, PA). Acquisition and hardware were controlled by MetaMorph 7.7 (Molecular Devices, Downingtown PA). A Hamamatsu ImagEM EMCCD camera (Bridgewater, NJ) was used for image capture. Diode lasers for excitation (488 nm for GFP and 561 nm for mCherry/RFP) were housed in a module constructed by Spectral Applied Research (Richmond Hill, Ontario). All components were integrated by BioVision Technologies (Exton, PA).

### Real Time Cell Electronic Sensing (RT-CES) assay

The RT-CES assay is based on electrical impedance readings in cell monolayers plated in multi-wells plates containing gold microelectrodes embedded in the bottom of each well. We have used the analyzer, 16 well e-plates, and the integrated software from Acea Biosciences Inc. (San Diego, California). Cells were plated in 100 µl of media to achieve monolayer confluency. The analyzer and the installed plates were placed in a standard cell culture incubator, at 37°C in a humidified atmosphere of 5% carbon dioxide. Cells were allowed to adhere to plates overnight and cell impedance recordings were performed during addition of drugs to the medium. Data was recorded and analyzed using the integrated software.

### Western Blot Analysis

Cells were lysed in lithium dodecyl sulfate (LDS) sample buffer (Invitrogen, Carlsbad, CA), and lysed using a sonic dismembrator model 100 (Fisher Scientific, Hampton, NH). Cell extracts were subjected to LDS polyacrylamide gel electrophoresis and transferred to polyvinylidene difluoride membranes. After blocking with 5% nonfat dry milk in 0.1% Tween 20/TBS, membranes were incubated with the primary antibody. The following phospho-specific antibodies were used: anti- myosin light chain 2 (MLC2) (Ser19) and anti-PAK1 (ser199/204)/PAK2 (ser192/197) (Cell Signaling Technology, Beverly, MA), α-tubulin (Tyr-272) (Epitomics, Burlingame, CA) and β-tubulin (Ser-172) (ECM Biosciences, Versailles, KY). The following non phospho-specific antibodies were used: Beta-Actin and PAK1/2/3 (Cell Signaling Technology, Beverly, MA). Secondary antibodies conjugated to horseradish peroxidase were either anti-rabbit (Biorad, Hercules, CA), or anti-mouse (Cell Signaling Technology, Beverly, MA). Bands were visualized by enhanced chemiluminescence using SuperSignal® West Pico Chemiluminescent Substrate (Thermo Scientific, Rockford, IL) on a VersaDoc Imager (BioRad, Hercules, CA).

### Statistical analysis

Three to six independent experiments were performed for each condition and mean values and standard errors were calculated. One way analysis of variance and a two-tailed t-test were used to determine the statistical significance of differences between means (* p<0.05).

## Supporting Information

Video S1 **Video microscopy recordings in U373MG cells stimulated with SP in the absence (top-left panel) or presence of the NK1R antagonist aprepitant (bottom-left panel), the PLC inhibitor U73122 (top middle panel), the Rho inhibitor C3 transferase (bottom-middle panel), the ROCK inhibitor Y27632 (top-right panel), and the MLCK inhibitor ML-9 (bottom-right panel), respectively.** The addition of SP (100 nM) and the time corresponding to each frame are indicated in the bottom-left corner.
(AVI)

Video S2 **Video microscopy recording showing morphology changes induced by SP in U373MG cells expressing tubulin-GFP, as described in** **figure 5** **.**
(AVI)

Video S3 **Video microscopy recording showing morphology changes induced by SP in HEK293-NK1R cells expressing tubulin-GFP and actin-RFP, as described in** **figure 5** **.**
(AVI)

Video S4 **Video microscopy recordings in U373MG cells (top panels) and HEK293-NK1R (bottom panels) stimulated with SP (10 nM) in the absence (left panels) or presence of colchicine (middle panels), and the PAK inhibitor IPA3 (right panels).** The addition of SP (100 nM) and the time corresponding to each frame are indicated in the bottom-left corner.
(AVI)
